# Simulating cellular galectin networks by mixing galectins in vitro reveals synergistic activity

**DOI:** 10.1016/j.bbrep.2021.101116

**Published:** 2021-08-28

**Authors:** Ruud P.M. Dings, Nigam Kumar, Sterling Mikkelson, Hans-Joachim Gabius, Kevin H. Mayo

**Affiliations:** a Department of Biochemistry, Molecular Biology & Biophysics, University of Minnesota, Minneapolis, MN, 55455, USA; b Institute of Physiological Chemistry, Faculty of Veterinary Medicine, Ludwig-Maximilians-University, Veterinarstr. 13, Munich, 80539, Germany

**Keywords:** Adhesion, Agglutinin, Apoptosis, Lectin, Migration, CRD, carbohydrate recognition domain, EC, endothelial cells, FITC, fluorescein isothiocyanate, Gal-1, galectin-1, Gal-3, galectin-3, Gal-7, galectin-7, MFI, mean fluorescence intensity, PS, phosphatidylserine, RBC, red blood cells

## Abstract

**Background:**

Even though members of the family of adhesion/growth-regulatory galectins are increasingly detected to be co-expressed, they are still being routinely tested separately. The recent discovery of heterodimer formation among galectins-1, -3, and -7 in mixtures prompts further study of their functional activities in mixtures.

**Methods:**

Cell agglutination, galectin binding to cells, as well as effects on cell proliferation, onset of apoptosis and migration were determined in assays using various cell types and mixtures of galectins-1, -3, and -7.

**Results:**

Evidence for a more than additive increases of experimental parameters was consistently obtained.

**Conclusion:**

Testing galectins in mixtures simulates the situation of co-expression *in situ* and reveals unsuspected over-additive activities. This new insight is relevant for analyzing galectin functionality in (patho)physiological conditions.

## Highlights

• Galectins-1, -3, and -7 form heterodimers in solution.
• Mixtures of galectins simulates galectin co-expression *in situ*.
• Mixtures display synergistic activities in vitro.
• Cell agglutination, apoptosis, proliferation, migration affected.
• Findings are relevant for galectin functionality *in vivo*.

## 1 Introduction

The realization of the enormous capacity of glycans of cellular glycoconjugates to serve as versatile molecular messages has directed interest to the study of endogenous lectins [[1], [2], [3]]. The specific glycoconjugate-lectin recognition is the first step to translate the encoded information into bioactivity. During this process, the modular architecture of lectins has emerged as an important factor, providing a rationale for the detected diversification on this level. In fact, the common carbohydrate recognition domain (CRD) is presented in various structural contexts in most lectin families.

**Fig. 1:**
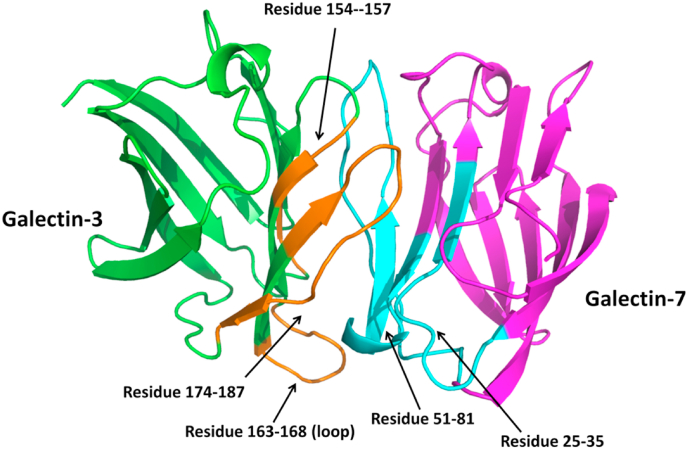
Model of the heterodimer formed by association of the CRDs of Gal-3 and -7 [9].

Looking at the adhesion/growth-regulatory galectins, vertebrates express these effectors in three types of architecture, i.e. non-covalently associated homodimers, linker-connected heterodimers, and a combination of the CRD with a different peptide section or module [[4], [5], [6]]. Because monitoring of galectin expression in cells and tissues reveals the occurrence of their co-expression [7,8], the question of potential functional cooperation becomes obvious. In addition, the hypothesis that mixing pairs of galectins acquires new structural permutations as hybrids has been tested, leading to the discovery of the formation of galectins heterodimers as structurally illustrated with the pairing of CRDs of galectins-3 and -7 (Gal-3 and -7, Fig. 1) [9]. The assumption that these new entities are biologically active is supported by the design of covalently-linked heterodimers of Gal-1 and -3 CRDs in which cell receptor capacity has been maintained [10].

It is now timely to explore the possibility of whether simulating *in situ* conditions by testing mixtures of galectins will affect galectin-mediated cell-based functions. Toward this end, we examined the outcome of functional assays (i.e. cell aggregation, growth regulation and migration) in which mixtures of Gal-1, -3 and -7 were used as a platform for hybrid formation.

## 2 Methods

### 2.1 Galectin production

Human galectins were obtained by recombinant production and affinity chromatography on Sepharose 4B resin presenting lactose as ligand. Purity, sequence identity and quaternary structure were ascertained by one- and two-dimensional electrophoresis, gel filtration and nano-electrospray ionization mass spectrometry, as described previously when detecting heterodimer formation [9].

### 2.2 Cells, cultures and reagents

Human umbilical vein endothelial cells (HUVECs) were harvested from normal human umbilical cords by perfusion with 0.125% trypsin/EDTA solution. HUVECs were cultured in gelatin-coated tissue-culture flasks (0.2%) in culture medium (RPMI 1640 with 20% (v/v) human serum, supplemented with 2 mM glutamine, 100 units/ml penicillin and 0.1 mg/ml streptomycin). 2H11 murine endothelial cells were cultured in flasks with uncoated surface using 10% fetal bovine serum and 1% penicillin/streptomycin in RPMI 1640 medium [[11], [12], [13], [14]]. Cultures were split every three days in 1:3 ratio.

### 2.3 Preparation of fluorescent galectin

Galectin was labeled with FITC using a FITC:protein molar ratio of 10:1 [15]. For this, lyophilized protein was dissolved in 500 μl of 20 mM potassium phosphate buffer, pH 7.4, to reach the final concentration of 2 mg/ml, followed by addition of ~50 μL FITC-containing solution (10 mg/ml) in 0.1 mM sodium bicarbonate. The resulting lower pH (~pH 3) ensured selective labeling at the N-terminal amine group of the protein [16,17]. This solution was then mixed thoroughly and incubated at room temperature (22 °C) for 18 h in the dark. During the course of the reaction, the mixture was gently vortexed three to four times. The resulting FITC-labeled protein was separated from unbound dye by extensive ultrafiltration using an Amicon Ultra cellulose filter (Millipore, 10 kDa cut-off). MALDI-TOF MS demonstrated the addition of the 389 Da FITC label to Gal-1 at a >90% labeling efficiency.

### 2.4 Agglutination assay

Murine red blood cells (RBCs) and human Jurkat E6.1 cells (2 × 10^6^/200 μL RPMI 1640 medium) were agglutinated upon addition of a solution containing 1 μM galectin. Cells were incubated in plastic round-bottom chamber slides (Nunc, Naperville, IL, USA) in medium alone or with medium containing galectins at 22 °C [15]. Subsequently, the extent of cell agglutination was measured at an absorbance of 650 nm, as described earlier [18].

### 2.5 Flow cytometry

Male and female Gal-1 null mice (Jackson Laboratory, Bar Harbor, ME, USA) were provided water and standard chow ad libitum and maintained on a 12-hr light/dark cycle prior to experiments that were approved by the University of Minnesota Research Animal Resources Ethical Committee. For FACS experiments, spleens from these mice (6–10 weeks old) were obtained surgically and non-enzymatically disrupted by shear force to yield single-cell suspensions [13,15]. These were prepared in Hanks's balanced solution. RBCs were lysed in ACK (Lonza Walkersville) for 5 min on ice, and suspensions were filtered through nylon mesh. Spleen cells were then washed and incubated with monoclonal antibodies as indicated for 40 min on ice. After an additional washing step, solution containing 1 μM FITC-Gal-1 was added to these cells in the absence or presence of various concentrations of other galectins. Mixtures were then incubated for 30 min on ice. Prior to FACScan analysis, cell suspensions were washed again and analyzed by multi-parameter flow cytometry on a LSR II flow cytometer (BD Biosciences) using FlowJo software (Tree Star, Inc.) [13,15]. For the dissociation rate assessment, splenocytes were used and processed as mentioned above, kept at 4 °C at all times. Per sample, 1 × 10^6^ cells were exposed to solution containing 2 μM FITC–Gal-1 (with or without 2 μM Gal-7) and, after a thorough washing step, mean fluorescence intensity (MFI) was measured over time in 2-min intervals as indicated.

### 2.6 Growth assays

Endothelial cells (2H11) were seeded in a 96-well culture plate coated with 0.2% gelatin for 2 h at 20 °C (Sigma-Aldrich, St. Louis, MO). Cells were seeded at a concentration of 3,000 cells per well and allowed to adhere for at least 3 h at 37 °C in 5% CO_2_/95% air before experiments were initiated. The cells were then exposed to complete medium with 20 ng/ml basic fibroblast growth factor (Sigma-Aldrich), with or without various concentrations of galectins for 72 h or as indicated otherwise. Cell counting kit (CCK-8; Dojindo, Gaithersburg, MD) was used to assess cell viability rates relative to untreated cells, as described earlier [[11], [12], [13],^19^]. All measurements were done in triplicate, and the experiments were done at least three times.

### 2.7 Apoptosis assay

Splenocytes were left untreated or treated with Gal-1, -3, and/or −7 at the indicated concentration. According to the manufacturer's instructions (R&D systems), annexin V(AnxV)-FITC was used to assess early apoptosis, and 7-aminoactinomycin D (7AAD) was used to assess late apoptosis and/or necrotic cell detection. To differentiate the effects on different cell types, splenocytes were stained for surface markers CD4 (clone RM4-5) labeled with Alexa Fluor 700 and CD8 (clone 53-6.7) labeled with Alexa Fluor 780, endothelial cells for CD31 (clone MEC 13.3) labeled with phycoerythrin (PE).

### 2.8 Migration assay

Endothelial cells (2H11) were seeded on 8-well culture plates and grown to confluency at 37 °C in 5% CO_2_/95% air. Confluent cells were then scrapped with a rubber spatula and were photographed at zero time and after 12 h incubation. The degree or percentage of wound closure was calculated by taking the open, unfilled area minus the area occupied by new cell growth within the scrapped area at the 12 h time point and dividing it by the open area at the zero-time point.

### 2.9 Statistical analysis

Data are reported as the mean ± SEM unless otherwise stated. Data were analyzed by using an unpaired, two-tailed *t*-test. *P* values < 0.05 were considered to be statistically significant.

## 3 Results

**Fig. 2:**
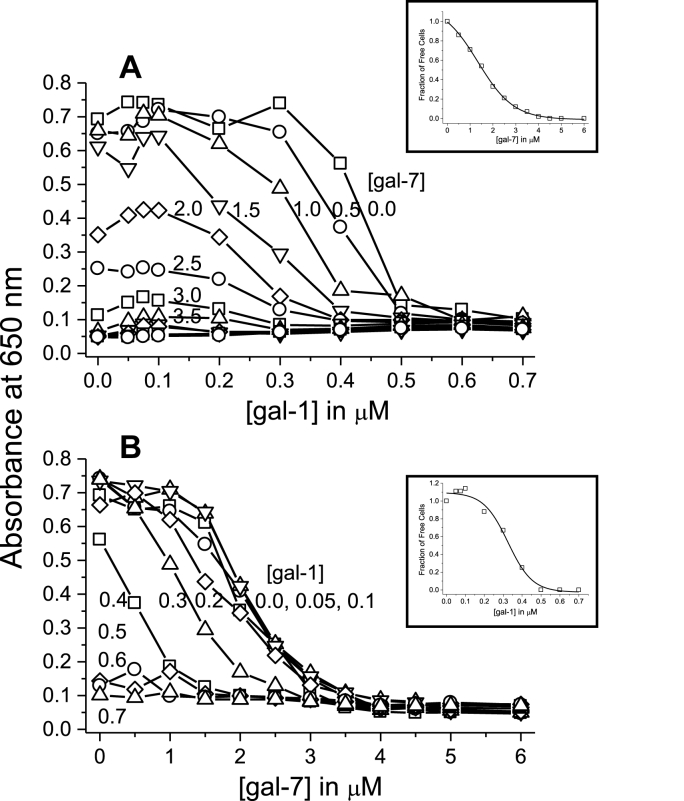
RBC agglutination. Using data shown in Supplemental Fig. S2 and a microtiter plate reader, erythrocyte agglutination was quantitated by measuring the absorbance at 650 nm, A_650_, plotted as a function of the concentration of Gal-1 (**A**) and Gal-7 (**B**). In each plot, the concentration of these paired galectins was varied from 0.05 to 0.7 μM for Gal-1 and from 0.4 to 1.8 μM for Gal-7, as indicated in the figure. Data are presented as the mean of four independent experiments with n = 2 per data point. SEM values for all data points are <9% and have been omitted in plots for clarity.

Agglutination studies are often used to assess galectin-mediated cell-cell adhesion. We discovered that when Gal-1 and Gal-7 are mixed in solution, they mediate cell agglutination synergistically. Fig. 2 shows the extent of red blood cell (RBC) agglutination (actual data shown in Supplemental Fig. S1) by plotting the absorbance at 650 nm, A_650_, as a function of Gal-1 and -7 concentrations. Gal-1 alone agglutinates RBCs at an EC_50_ = 0.45 μM, whereas Gal-7 alone is less potent with an EC_50_ = 2.2 μM. Upon mixing Gal-1 and -7 at various concentrations (i.e. Gal-1 from 0.05 to 0.7 μM and Gal-7 from 0.4 to 1.8 μM), agglutination was observed to occur at lower total concentrations compared to experiments with either galectin alone (Fig. 2). In this instance, 50% agglutination was obtained with a mixture of 0.25 μM Gal-1 and 1.2 μM Gal-7, or about half the concentration required with each galectin alone.

In further support of this finding, we used a mutant of Gal-1 that has been reported to primarily form monomers (Gal-1m) [20,21]. To confirm that we had a mostly monomer population, we performed PFG NMR experiments to determine diffusion coefficients, *D*, for Gal-1m [22]. The *D* value for native dimeric Gal-1 is 1.04 × 10^−6^ cm^2^/s [22], whereas the *D* value for Gal-1m at the highest concentration used in our agglutination studies is ~0.7 × 10^−6^ cm^2^/s, a value consistent with a >95% monomeric population of Gal-1m [22]. With this in mind, we found that neither Gal-1m nor Gal-7 alone (both monomeric at the concentrations investigated) induced agglutination (Supplemental Fig. S2). However, when these two galectins were combined at specific concentrations, agglutination became significant (Supplemental Fig. S2), indicating that these two galectins indeed function in concert. In line with the galectin cross-linking model, our data strongly suggest that the two galectins physically interact with each other to promote agglutination, rather than interacting with e.g. separate glyco-conjugate receptors on interacting cells. In addition, we also performed agglutination experiments with individual galectins in combination with human serum albumin (HSA), and found that HSA has no influence when mixed with these galectins.

Gal-1 and -7 also function in concert to promote leukocyte (Jurkat) cell agglutination (Supplemental Fig. S3). Gal-1 alone induces leukocyte agglutination at and above 0.2 μM, whereas Gal-7 alone does so at 2.0 μM (Supplemental Fig. S3A). Combinations of Gal-1 and Gal-7 promote leukocyte agglutination in a greater than additive fashion. In this instance, agglutination was observed at less than half their individual effective concentrations (e.g. at 0.06 μM Gal-1 and 0.6 μM Gal-7). This apparent synergistic effect is shown in Supplemental Fig. S3B, where agglutination is induced at less than half their individual effective concentrations (e.g. at 0.06 μM Gal-1 and 0.6 μM Gal-7). Overall, these studies indicate that Gal-7 and Gal-1 in combination function dependently in cell agglutination. As with our RBC agglutination experiments, studies with mixtures of Gal-1m and Gal-7 support this conclusion (data not shown).

**Fig. 3:**
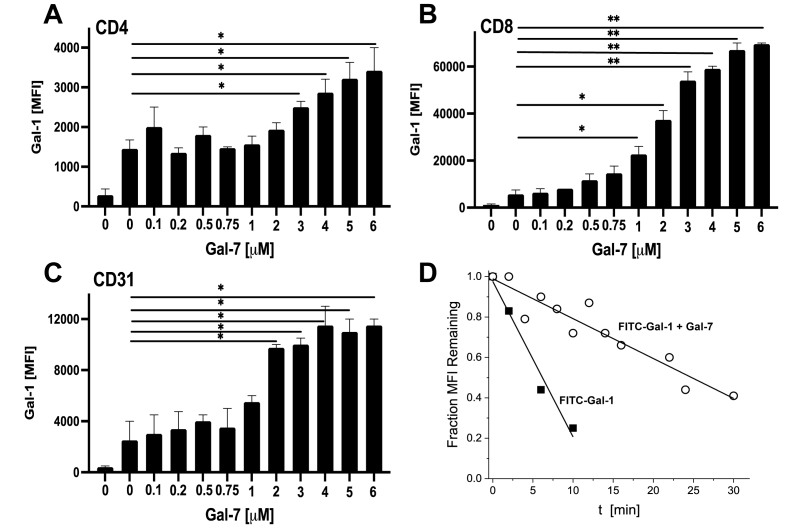
FACS data of binding of fluorescent FITC-labeled Gal-1 to mouse splenocytes. Solution with fluorescent Gal-1 (0.5 μM) was used to stain mouse splenocytes in the presence of varying concentrations of label-free Gal-7 from 0.1 μM to 6 μM. Splenocytes were stained for CD4 (**A**) and CD8 leukocytes (**B**), CD31^+^ endothelial cells were also tested (**C**). MFI is shown on the y-axis. Experiments were performed at 4 °C. (**D**) Rates of dissociation of FITC–Gal-1 from the cell surface. Splenocytes (1 × 10^6^ cells per sample) were stained with 2 μM fluorescent Gal-1 (with or without 2 μM Gal-7) and, after a thorough washing step to remove free marker, MFI was measured as a function of time in ~2 min intervals. Data are presented as the mean fluorescence intensity (MFI) ± SEM and are representative of three independent experiments with n = 2 per data point. *P < 0.05, **P < 0.01 using the two-tailed *t*-test.

Because galectins function extracellularly by binding glycans on the cell surface, we performed FACS analyses to assess galectin binding to cells. For this, we used FITC-labeled Gal-1 with fresh mouse splenocytes stained for CD4^+^ and CD8^+^ (leukocytes) and for CD31^+^ (endothelial) cells. Single cell solutions were stained with 0.5 μM FITC-Gal-1, and the Mean Fluorescence Intensity (MFI) which is proportional to the amount of FITC-Gal-1 bound per cell, was measured. FITC-Gal-1 binding is greatest to glycans on endothelial (CD4-/CD8-/CD31+) cells (MFI 3392) and less to glycans on CD8^+^ (MFI 1867) and CD4^+^ (MFI 1945) leukocytes (Fig. 3A–C), a finding that most likely relates to differences in the number of Gal-1 binding sites and/or binding affinity between cell types. With each of these cell types, addition of Gal-7 was observed to enhance FITC-Gal-1 binding in a concentration dependent manner (Fig. 3A–C) with increased binding being most evident with CD8^+^ cells (Fig. 3B).

In addition, we found that the initial dissociation rate of cell-bound FITC-Gal-1 was reduced in the presence of Gal-7 (1:1 M ratio) (Fig. 3D). Assuming a first-order reaction, we found that the half-life of association for FITC–Gal-1 used alone is ~6 min, whereas in the presence of Gal-7 it increased markedly to ~26 min. Because of the way in which this experiment was performed (see Methods Section), re-binding events should be minimal, especially at early time points. Moreover, because these half-life values are directly related to the off-rate, differences in the number of binding sites would play no role. Thus, binding properties of one galectin is positively affected by the presence of another. This finding supports the idea that these galectins function in concert and is consistent with our observation of enhanced cell agglutination.

**Fig. 4:**
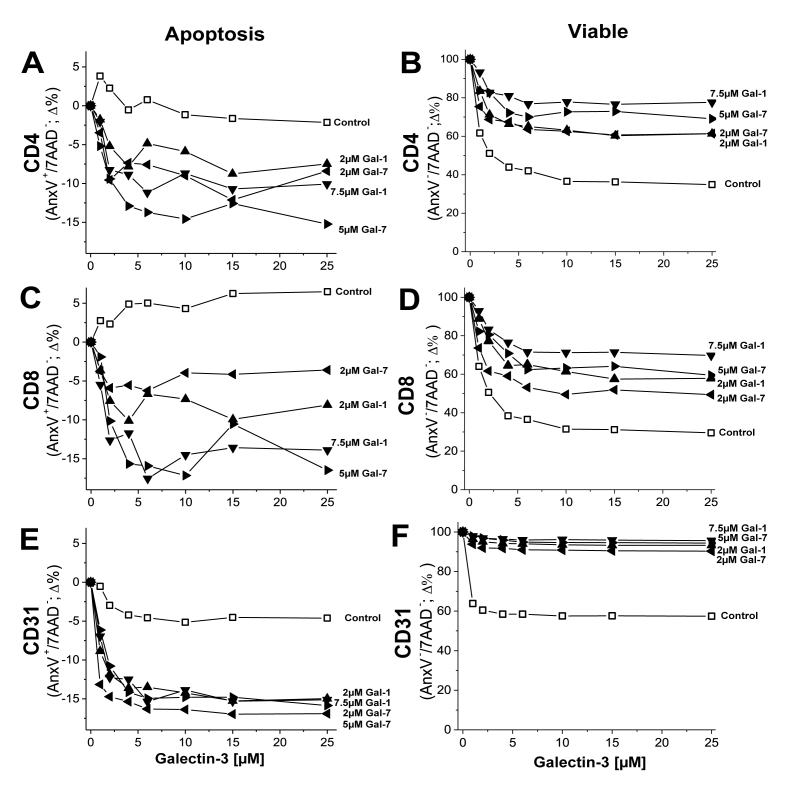
The concentration dependence for full-length Gal-3 on the percentage of cells (mouse splenocytes) stained with labeled annexin V (**A**,**C**,**E**) to assess early-stage apoptosis, and with the antibody 7AAD (**B,D,F**) to gate on dead cells and assess the number of viable cells, as discussed in the text. Splenocytes were also stained for surface markers CD4 (**A**,**B**) and CD8 (**C**,**D**) (leukocytes) and endothelial cells were labeled by fluorescent CD31 (**E**,**F**). Data are shown for Gal-3 alone (control) and Gal-3 in the presence of fixed concentrations of Gal-1 (2 μM and 7.5 μM) and Gal-7 (2 μM and 5 μM). Data are presented as the mean and are representative of three independent experiments with n = 2 per data point. SEM values for all points are <9% and have been omitted from plots for clarity.

Because galectins can also induce cell apoptosis and cell death [[23], [24], [25]], we investigated whether mixed galectins could modulate these activities. For this, we used combinations of Gal-1, -3 and -7 in FACS studies to assess early stage apoptosis (Annexin V, AnxV^+^ population [26]) and cell viability (7AAD^−^ population with 7AAD^+^ staining to gate on dead cells) in mouse splenocytes stained for CD4^+^, CD8^+^, and CD31^+^ cells. Fig. 4 shows results for the concentration dependence of Gal-3 in the presence of a fixed concentration of Gal-1 (2 μM and 7.5 μM) and Gal-7 (2 μM and 5 μM). As the concentration of Gal-3 alone is increased, the percentage of cells in early stage apoptosis (AnxV^+^/7AAD^−^) changes minimally in CD4^+^ (Fig. 4A) and CD31^+^ (Fig. 4E) cells, whereas it increases slightly in CD8^+^ cells (Fig. 4C). On the other hand, the percentage of live cells (AnxV^−^/7AAD^−^) decreases significantly in all three cell types over the same Gal-3 concentration range (Fig. 4B,D,F). Accordingly, the percentage of dead cells (AnxV^−^/7AAD^+^) rises (data not shown). These findings are consistent with previous reports, where it was shown that Gal-3 induces e.g. macrophage-mediated cell death vis-à-vis apoptosis [27,28].

In the presence of Gal-1 or Gal-7, effects from Gal-3 are different. Overall, the mixture of these galectins significantly reduces the relative percentage of Gal-3-induced apoptosis (Fig. 4A,C,E) and increases the population of viable cells (Fig. 4B,D,F). Moreover, there is an apparent dose response from the presence of different concentrations of Gal-1 and Gal-7, i.e. the higher the concentration, the greater the effect on Gal-3 function. Since the percentage change is greatest overall with CD31^+^ cells, the dose response here is either absent or less apparent. The extent of galectin-induced onset of apoptosis was assessed by measuring the percentage of cells exposing phosphatidylserine (PS) on their surface (a common assay used when testing galectins [29]) (Supplemental Fig. S4). Gal-1 and Gal-7 alone induce significant early stage apoptosis (Supplemental Figs. S4A and C), consistent with previous reports [30]. As a function of the concentration of Gal-1 or -7 in the absence (Supplemental Figs. S4A and C, respectively) and presence of Gal-7 (3 μM, Supplemental Fig. S4B) or Gal-1 (7.5 μM, Supplemental Fig. S4D), we found that the percentage of PS-exposing cells was significantly reduced when Gal-1 and -7 were mixed, more so when the concentration of Gal-7 was fixed and that of Gal-1 was varied.

**Fig. 5:**
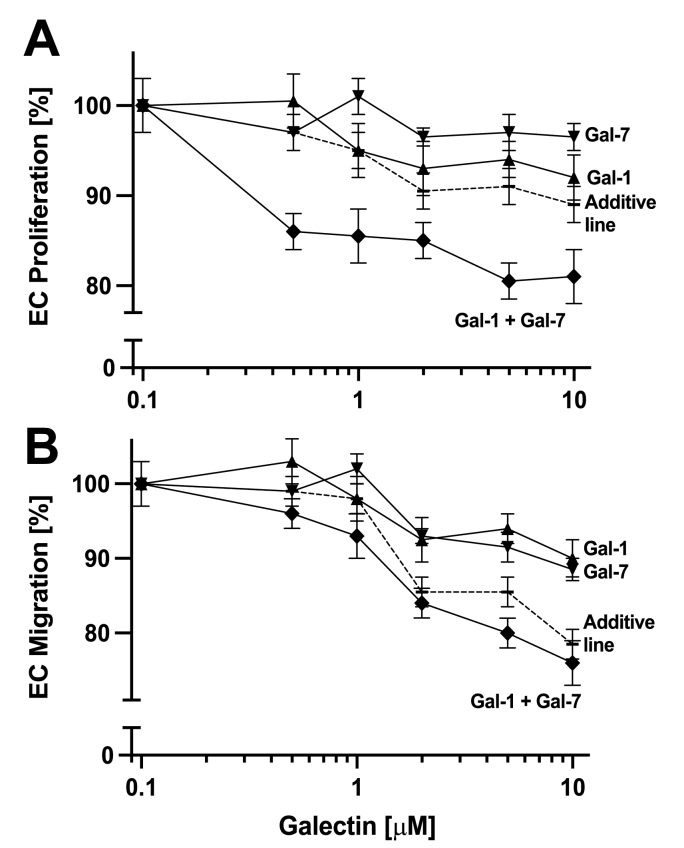
Effects from mixtures of Gal-1 and -7 on endothelial cell proliferation (**A**) and cell migration (**B**) are shown. In the proliferation assay, mouse endothelial cells (2H11) were cultured in the presence of Gal-1 (10 μM), Gal-7 (10 μM) or a combination of the both proteins (5 μM + 5 μM). After three days of cell culture, the number of viable cells was measured by colorimetric read-out and normalized to wells with untreated cells. In the wound healing/cell migration assay, a confluent layer of endothelial cells was scrapped using a rubber spatual, and wound closure was monitored after 12 h (raw data shown in Supplemental Fig. S5). Data are presented as the mean ± SEM and are representative of three independent experiments with n = 3 per data point in panel A and n = 2 in panel B. The ‘additive line’ is the mathematically predicted curve based on the sum of the activities of individual galectins.

Galectins can also influence endothelial cell proliferation and migration [25]. Fig. 5A shows the percent of mouse 2H11 endothelial cell proliferation relative to control vs. the concentration of Gal-1 and Gal-7 alone (reported previously to be small [25]) and in a 1:1 M ratio (same total concentration). However, the combination of Gal-1 and Gal-7 attenuates cell growth compared to either galectin alone (Fig. 5A), an effect that is most evident at higher galectin concentrations. The same is observed with EC migration (Fig. 5B). In this wound healing assay (raw data shown in Supplemental Fig. S5), Gal-1 or Gal-7 alone decreased the rate of wound closure. Over the time frame of 12 h, the combination of 5 μM Gal-1 and 5 μM Gal-7 effectively reduced the extent of EC migration by about 23%, whereas with either galectin alone at 10 μM, the effect was only about 10%. Thus, the activities of these two galectins in combination are synergistic.

## 4 Discussion

Functional analysis of galectins has taken great strides by testing proteins individually, with a focus on Gal-1 and -3. Since systematic expression profiling is teaching us the lesson of co-expression of galectins building a network, for example in embryogenesis [31] or in the pathogenesis of osteoarthritis [32], we reasoned that i) assays with galectins in combination may reveal evidence for cross-talk among these proteins to understand the full spectrum of their physiological significance, and ii) the documented possibility of heterodimer formation may have a functional impact.

The results presented in this report bring to light greater than additive effects when testing specific galectins known to engage in heterodimer formation in binary combinations. Evidently, a local co-expression, as simulated by our mixtures, may well elicit such so far unexpected consequences, hereby providing a new direction to further research. On the structural level, the availability of galectin CRDs (by proteolytic truncation for Gal-3 or a molecular switch for Gal-7 [33]) and the dynamic *in situ* generation of galectin heterodimers favor assembly of new functional galectin entities in distinct microenvironments, e.g. in inflamed tissue rich in protease activity to turn full-length Gal-3 into the Gal-3 CRD. What at first may have sounded far-fetched (i.e. to consider co-expression as a playground for realizing new modular permutations) has recently received a structural basis from NMR- and MD-based studies [9].

Overall, the use of cell-based assays in the present study indicates that pairwise mixtures of Gal-1, Gal-3, and Gal-7 synergistically modulate galectin-mediated function. Two explanations are plausible: 1) each galectin in the pair binds to its own glyco-conjugate “receptor” on the cell surface to elicit a concerted response, or 2) the pair of galectins forms hetero-oligomers which function as a unit. We favor the later explanation, because it parallels our earlier reports on CXC and CC chemokines in which these effector molecules interact physically and function as heterodimers [[34], [35], [36], [37], [38], [39]]. Therefore, the network expression of galectins, like chemokines before them, leads to functional cooperation. When present as mixtures *in vivo*, heterodimers are likely to form under certain circumstances and thereby become a new topic for functional analysis, especially if the parental galectins (such as Gal-1, -3 or -7) trigger distinct effects or cascades toward a certain cellular response. Proceeding from engineering of covalently-linked (non-dissociable) homodimers of proto-type galectins [40] to producing the corresponding panel of heterodimers for Gal-1 and -7, is therefore in progress in our laboratories.

## Declaration of competing interest

The authors declare that they have no conflict of interests.
