# Supplementary material for: Simulating cellular galectin networks by mixing galectins in vitro reveals synergistic activity
Source: Biochem Biophys Rep. 2021 Aug 28;28:101116. doi: 10.1016/j.bbrep.2021.101116 (PMC8408429; doi:10.1016/j.bbrep.2021.101116)
Supplement: Multimedia component 1 [file mmc1.pdf]

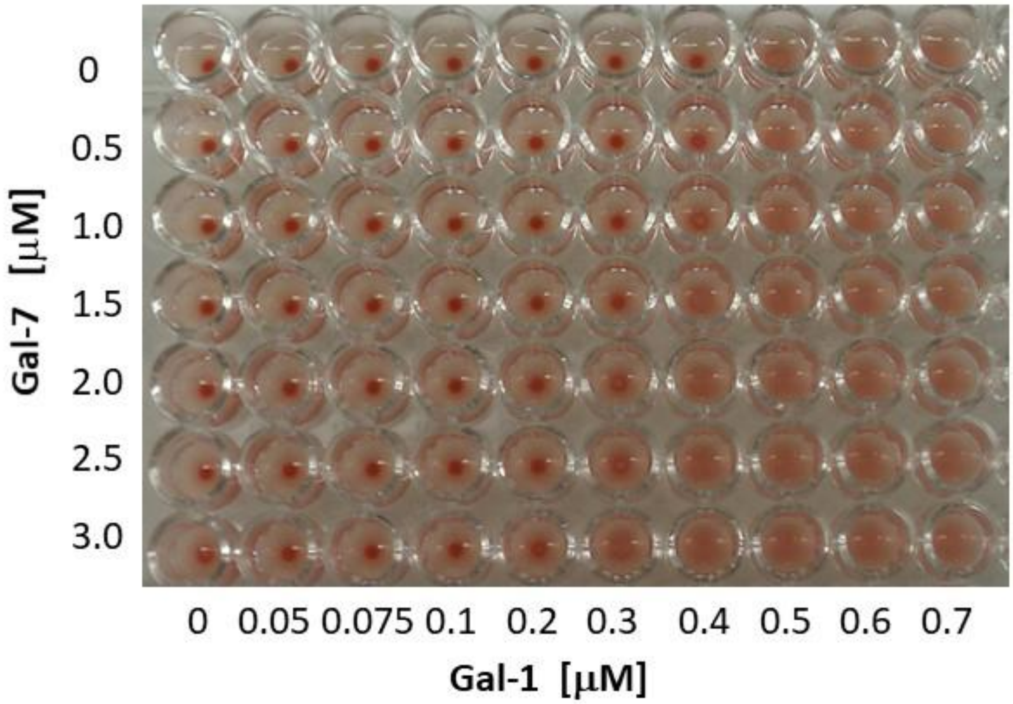

Supplemental Figure S1. RBC agglutination studies. Mouse RBCs were incubated in galectin-containing solutions as a function of various Gal-1 and Gal-7 concentrations as indicated. The first row at the top of the plate shows what happens as the concentration of Gal-1 alone is increased from zero to 0.7 μM, the first column at the left as the concentration of Gal-7 alone is increased from zero to 3 μM. Each well in the microtiter plates contains 2 x 10^6 erythrocytes from a Gal-$1^{-/-}$ null mouse and various concentrations of Gal-1 and -7. When cells do not agglutinate, they settle to a red "dot" at the bottom of a well, and when cells are completely agglutinated, the bottom of the well shows a diffuse region of cells.

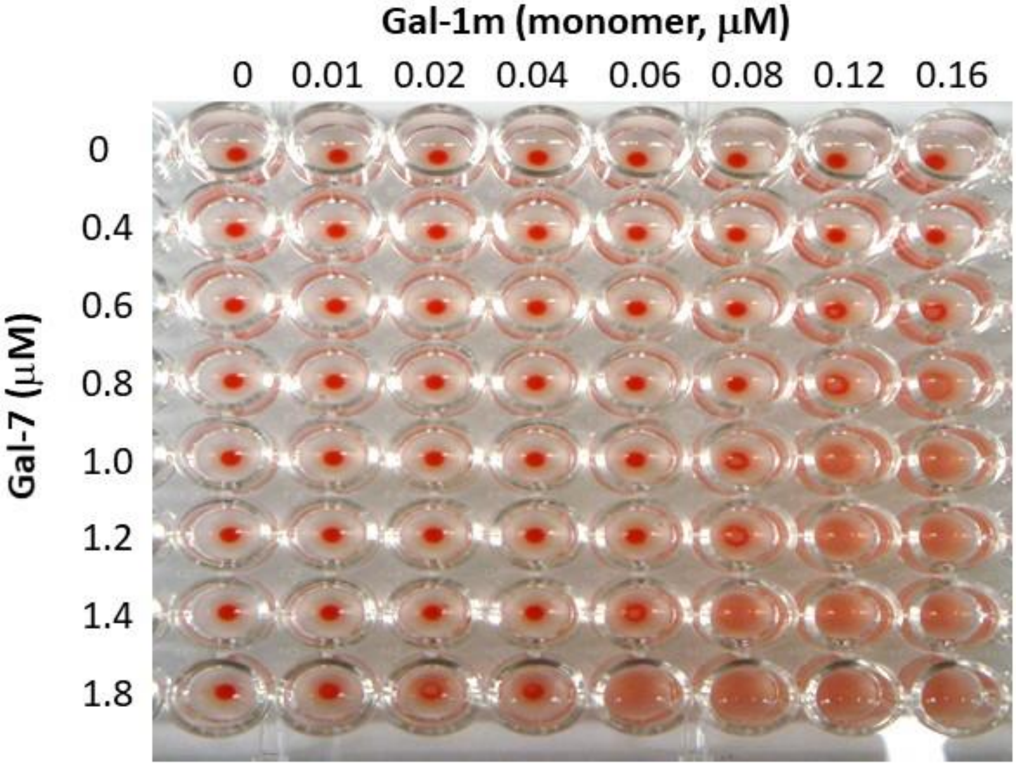

Supplemental Figure S2. RBC agglutination studies. Mouse RBCs were incubated in galectin-containing solutions as a function of various Gal-1m and Gal-7 concentrations as indicated. The first row at the top of the plate shows what happens as the concentration of Gal-1m alone is increased from zero to 0.16 μM, the first column at the left as the concentration of Gal-7 alone is increased from zero to 1.8 μM. Each well in the microtiter plates contains 2 x 10^6 erythrocytes from a Gal-$1^{-/-}$ null mouse and various concentrations of the galectin. When cells do not agglutinate, they settle to a red "dot" at the bottom of a well, and when cells are completely agglutinated, the bottom of the well shows a diffuse region of cells.

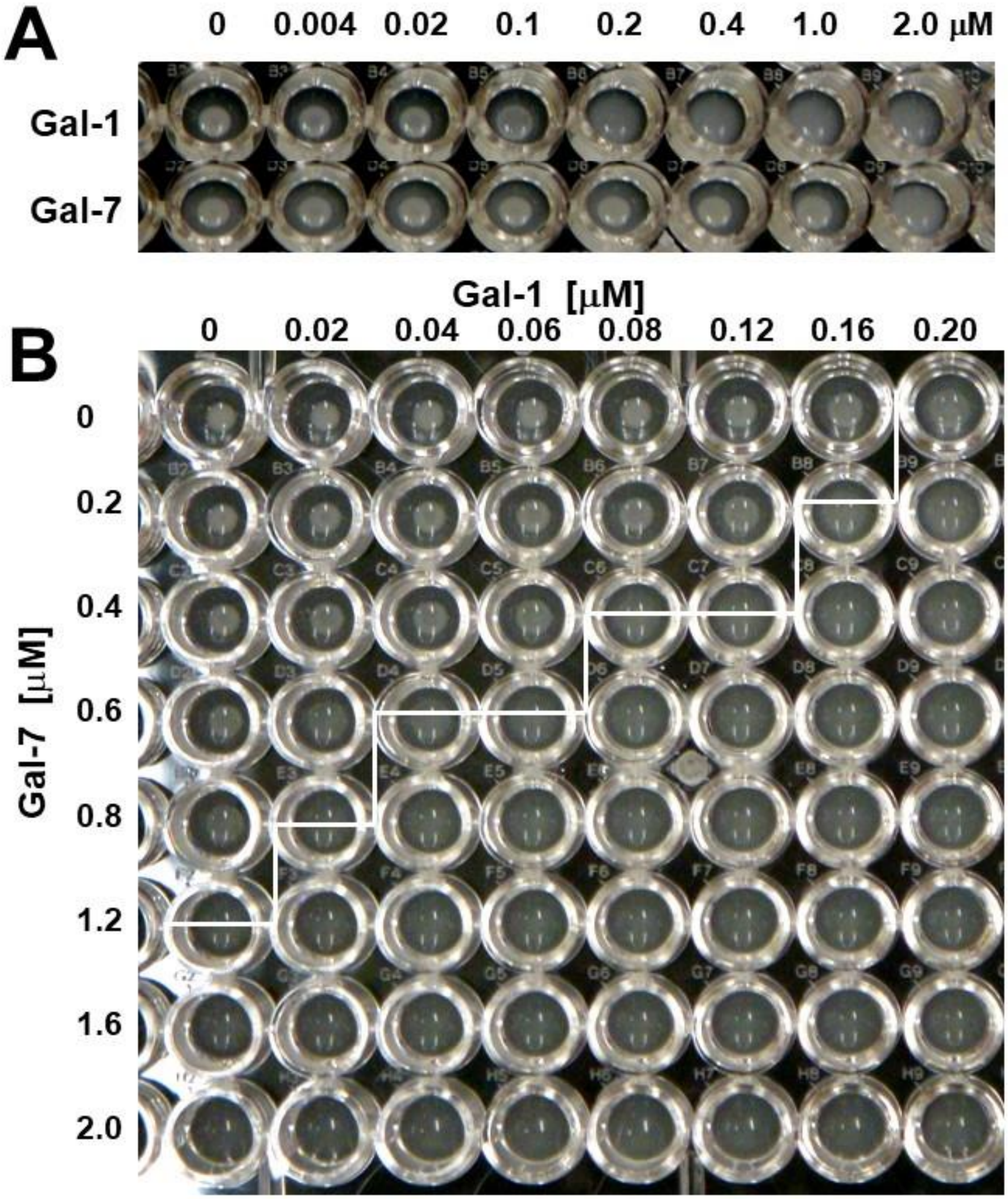

Supplemental Figure S3. Leukocyte agglutination. Jurkat cells incubated as function of 0 to 2 μM) Gal-1 or Gal-7. Each well has 2 x 10^6 cells incubated as a function of Gal-1 & -7 concentrations indicated. First row at top shows Gal-1 alone from 0 to 0.2 μM, and first column at left shows Gal-7 alone increased from 0 to 2 μM. White line shows where cells are observed to agglutinate.

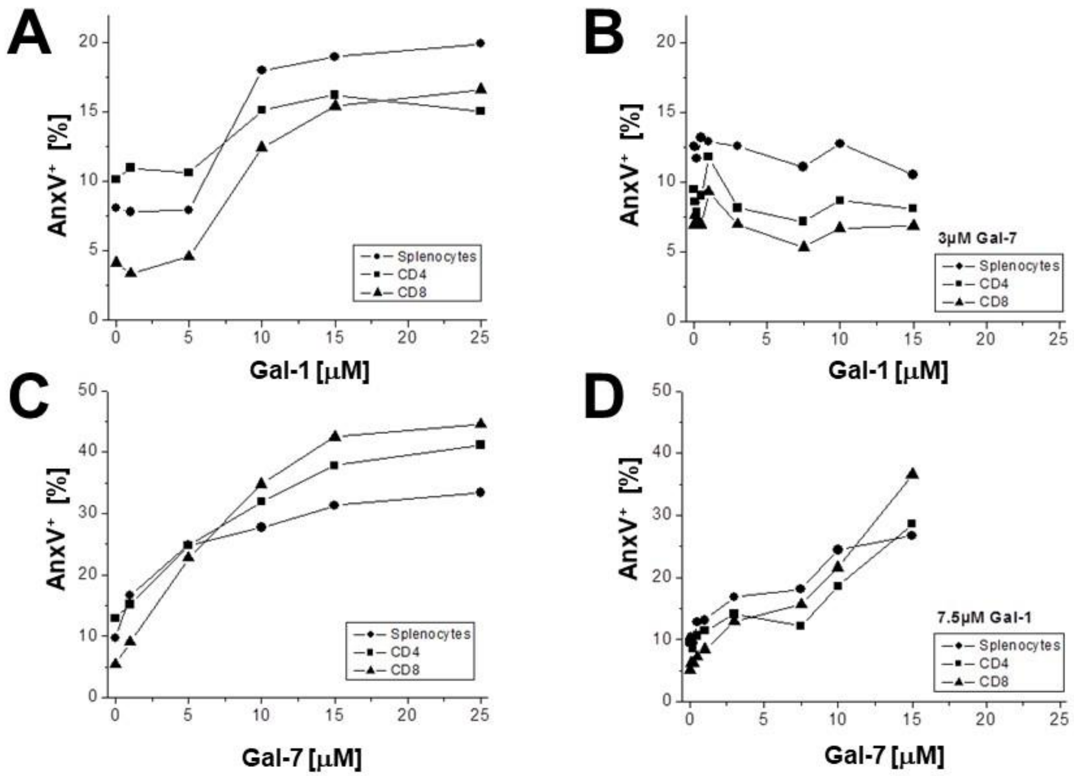

Supplemental Figure S4. Percentages of PS-presenting splenocytes (annexin V staining) are shown as a function of the concentration of Gal-1 (**A,B**) and Gal-7 (**C,D**). Splenocytes were stained for CD4 and CD8 (leukocytes), and endothelial cells for CD31, as indicated in the figure. Data are shown in the absence (**A,C**) and the presence of 3 μM Gal-7 (**B**) or 7.5 μM Gal-1 (**D**).

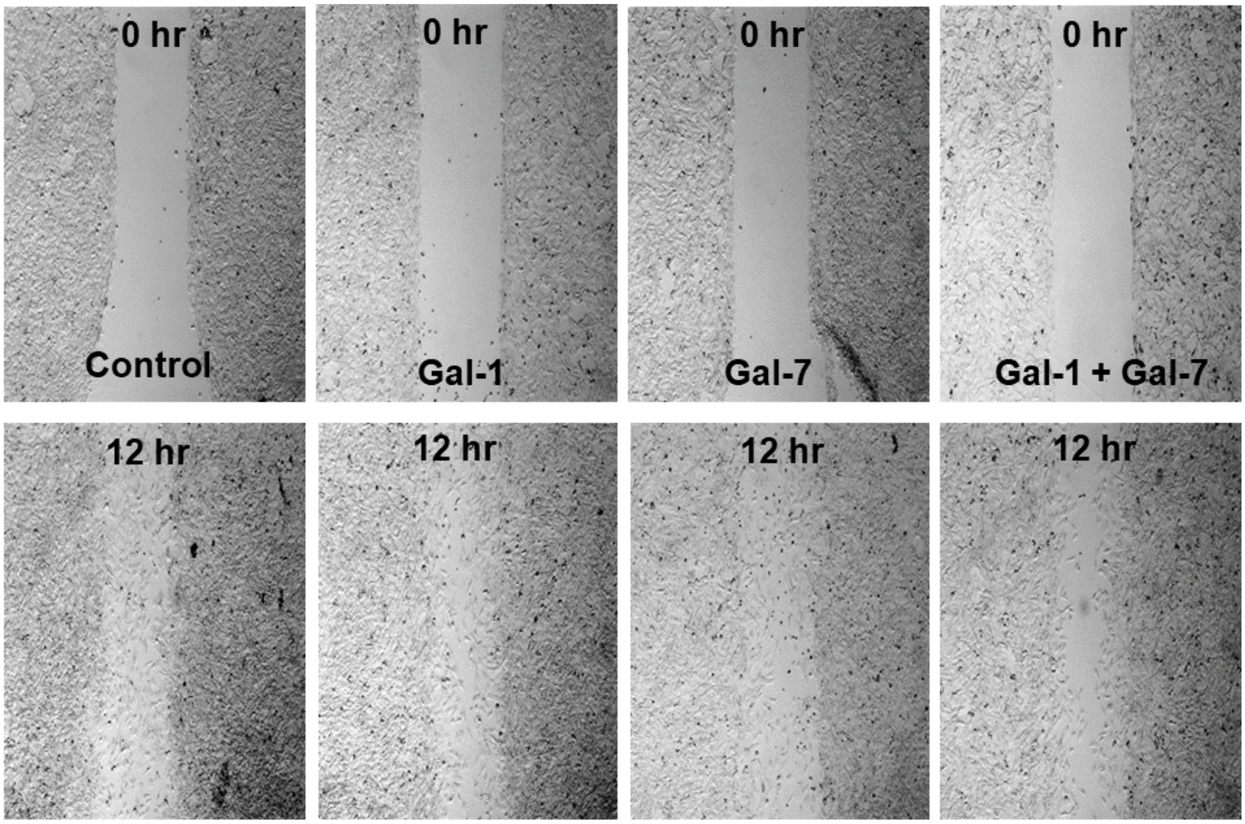

Supplemental Figure S5. Wound-healing assay. Confluent mouse EC scrapped, and closure monitored at 12 hrs. Control (media) and Gal-1 & -7 alone at 10 μM or mixed at 5 μM each.
